# Identification of the Interactions Interference Between the PH and START Domain of CERT by Limonoid and HPA Inhibitors

**DOI:** 10.3389/fmolb.2020.603983

**Published:** 2020-11-27

**Authors:** Mariem Ghoula, Axelle Le Marec, Christophe Magnan, Hervé Le Stunff, Olivier Taboureau

**Affiliations:** ^1^Université de Paris, INSERM U1133, CNRS UMR 8251, Paris, France; ^2^Université de Paris, BFA CNRS UMR 8251, Paris, France; ^3^Université Paris Saclay, Institut des Neurosciences Paris Saclay, CNRS UMR 9197, Orsay, France

**Keywords:** CERT, START domain, PH domain, limonoid inhibitors, cancer therapy, Ceramide

## Abstract

The multi domain ceramide transfer protein (CERT) which contains the domains START and PH, is a protein that allows the transport of ceramide from the endoplasmic reticulum to the Golgi and so it plays a major role in sphingolipid metabolism. Recently, the crystal structure of the PH-START complex has been released, suggesting an inhibitory action of START to the binding of the PH domain to the Golgi apparatus and thus limiting the CERT activity. Our study presents a combination of docking and molecular dynamic simulations of N-(3-hydroxy-1-hydroxymethyl-3-phenylpropyl)alkanamides (HPA) analogs and limonoids compounds known to inhibit CERT. Through our computational study, we compared the binding affinity of 14 ligands at both domains (START and PH) and also at the START-PH interface, including several mutations known to play a role in the CERT’s activity. At the difference of HPA compounds, limonoids have a stronger binding affinity for the START-PH interface. Furthermore, 2 inhibitors (HPA-12 and isogedunin) were investigated through molecular dynamic (MD) simulations. 50 ns of molecular dynamic simulations have displayed the stability of isogedunin as well as keys residues in the binding of this molecule at the interface of the PH-START complex. Therefore, this study suggests a novel inhibitory mechanism of CERT for limonoid compounds involving the stabilization of the START-PH interface. This could help to develop new and potentially more selective inhibitors of this transporter, which is a potent target in cancer therapy.

## Introduction

Sphingolipids belong to a major class of lipids in eukaryotic cells. They are not only involved in the membrane structure. They also act as important mediators in cellular signaling (Hannun and Obeid, 2008). Sphingolipid metabolism is highly regulated by various enzymes located in different subcellular compartments (i.e., endoplasmic reticulum, Golgi apparatus, plasma membrane, mitochondria, and lysosomes). This compartmentalized enzymatic network contributes largely to the cellular function of sphingolipids. Among these sphingolipids, ceramides have been shown to play a central role in the induction of apoptosis (Hannun and Obeid, 2018) and several ceramide metabolizing enzymes have been involved in induced-apoptosis in response to a variety of agents such as cytokines, chemotherapy and radiotherapy (Reynolds et al., 2004; Nganga et al., 2018). In contrast, other sphingolipids derived from ceramide metabolism, such as sphingosine-1-phosphate (S1P), sphingomyelin and glucosylceramide, have been shown to play either proliferative or protective properties (Hannun and Obeid, 2008; Maceyka et al., 2012). Cancer cells seem to have an altered balance between pools of sphingolipids promoting tumors from those having a suppressing role (Furuya et al., 2011; Morad and Cabot, 2013) which finally favor cells proliferation/survival. The inability of cancer cells to accumulate pro-apoptotic ceramides can be the consequence of not only a defect of *de novo* biosynthesis but also of an increased degradation into S1P or transformation of ceramide into sphingomyelin/glucosylceramide (Liu et al., 2013; Yandim et al., 2013). Consequently, the inability to accumulate ceramides has been associated with insensitivity to apoptosis induced by chemotherapy and radiotherapy (Dimanche-Boitrel and Rebillard, 2013). Importantly, inhibition of ceramide metabolism into glucosylceramide has been shown to be a critical factor to restore ceramide and re-sensitizes cancer cells to chemotherapy (Morad and Cabot, 2013).

Looking at the *de novo* ceramide biosynthesis which takes place in the endoplasmic reticulum (ER), it is known that the ceramides conversion into complex sphingolipids is not only based on enzyme activities. Namely glucosylceramide and sphingomyelin synthases (Gault et al., 2010). They are also regulated by specific transport between the ER and the Golgi apparatus (Kumagai and Hanada, 2019). Indeed, *de novo* sphingomyelin biosynthesis relies on non-vesicular ceramide trafficking of ceramide mediated by the ceramide transporter protein CERT. Specifically, CERT transports ceramides from the ER to the trans-Golgi regions at the ER–Golgi membrane contact sites. The inactivation of this transporter was shown to be a cellular response to induced apoptosis in several cell lines (Charruyer et al., 2008; Chandran and Machamer, 2012). It has been found that CERT expression is higher in drug-resistant cell lines (Swanton et al., 2007) and that molecular inhibition of CERT resulted in re-sensitization of cancer cells to chemotherapy (Lee et al., 2012; Palau et al., 2018). Taken together, this suggests that pharmacological inhibition of CERT can represent a novel anti-cancer strategy by overcoming drug resistance.

**FIGURE 1:**
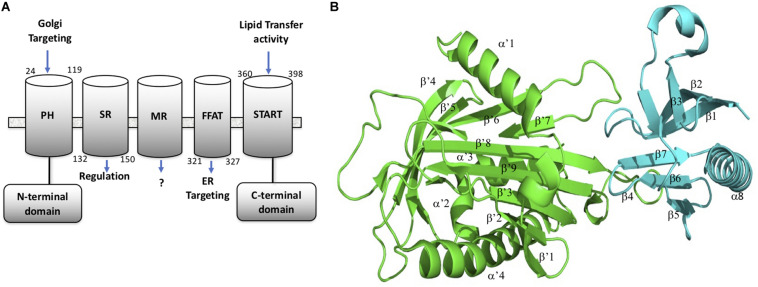
CERT domains and structure. **(A)** CERT domains and motifs. **(B)** PH (cyan) and START (green) domains representation in 3D structure from the 5JJD.pdb crystal structure. Secondary structures are annotated with α and β in PH domain and α’ and β’ in START domain, respectively.

CERT is a cytosolic monomeric protein constituted of different domains involved in its function (Kumagai and Hanada, 2019). CERT has a lipid binding START (STeroidogenic Acute Regulatory protein-related lipid Transfer) domain at the C-terminus, a PH (Pleckstrin homology) domain at the N-terminus, and a FFAT (diphenylalanine in an acidic track) in the middle region. The latter domain binds the ER-localized protein, VAP-A, whereas the START domain is responsible for the binding and transport of ceramide (Hanada et al., 2003). The PH domain also plays a role in ceramide transport by binding the phosphoinositide, phosphatidyl-inositol-4-phosphate (PtdIns4P) which is abundant in the Golgi apparatus (De Matteis et al., 2005). START domain, PH domain, and FFAT motif are all required for the full activity of CERT. CERT has also a serine-repeat (SR) motif, which decreases PtdIns4P binding and ceramide transfer activity when it is phosphorylated. It has been shown that the inhibition of PtdIns4P binding to the PH domain by hyper-phosphorylated SR motif requires the presence of the START domain (Kumagai et al., 2007). Recent crystal structure revealed that in fact the START domain interacts with PH domain at its PtdIns4P binding site (Prashek et al., 2017). Amino acid mutations that disrupt the PH/START interaction, increase ceramide-transfer activity of CERT, suggesting that this interaction plays an important role in the regulation of CERT cellular localization and ceramide transfer. The PH domain is formed of one α helix (α8) and seven β-sheets (β1– β7). The START domain is structured with four α helix (α’1– α’4) and nine β-sheets (β’1– β’9) Representation of the CERT domains and START-PH interaction complex are summarized in Figure 1.

Three unrelated families of CERT inhibitors have been described up to date. The N-(3-hydroxy-1-hydroxymethyl-3-phenylpropyl)alkanamides (HPA) family, synthesized analogs of ceramide, with HPA-12 as a lead, were identified as inhibitors of ceramide trafficking at the beginning of 2000 (Yasuda et al., 2001) by specifically binding to the START domain. It was found that the amphiphilic cavity of the START domain consists of hydrophobic residues that recognize the amide and hydroxyl groups of HPAs by hydrophobic interactions (Kudo et al., 2008). Importantly, many hydrophobic interactions are conserved in both HPA and ceramide binding, supporting a competitive inhibitory effect of this compound. More recently, the virtual screening of small chemicals leads to the discovery of a new CERT inhibitor, HPCB-5, which selectively binds the START domain (Nakao et al., 2019). At the difference of HPA compounds, HPCB-5 has no apparent ceramide mimicry and can considered as a ceramide-non-mimetic inhibitor of CERT. Another screen of a library of natural small compounds reveals that limonoids such as isogedunin selectively inhibited CERT activity (Hullin-Matsuda et al., 2012). It was shown that limonoids inhibit the CERT-mediated ceramide extraction from isolated ER membranes in order to block sphingomyelin biosynthesis. At the difference of HPA ligands, limonoids are unable to inhibit the rapid transfer of an exogenously added fluorescent short chain ceramide analog, supporting the idea of different inhibitory mechanisms of CERT mediated by HPA and limonoid families.

Up to date, there is no molecular mechanism proposed for the inhibitory effect of limonoid on CERT activity. The objective of this study is to determine the mechanism of interaction of limonoids to CERT in comparison to the binding of HPA ligands. We specifically explored the role of the new CERT regulation system where the PH and START domains interact with each other on a basal state to maintain CERT as inactive (Prashek et al., 2017). Accordingly, a computational study has been conducted to investigate the stability of isogedunin, and HPA-12 binding at the interaction interface of the two domains in order to maintain a self-inhibition.

First, a docking approach has been performed with a set of 16 ligands on the START, PH and START-PH complex system showing a preference of limonoid compounds to bind at the START-PH interaction site. Then, molecular dynamic (MD) simulations were performed to evaluate the stability of HPA-12 and isogedunin ligand at the interface of both domains and in a presence of mutations W33A, R43A, and Y54A at the PH domain and, E494R, N495K, P535R, and E537K at the START domain. These mutations have been described to be involved in the START-PH interaction (Prashek et al., 2017) and seem to also play a role in the interaction of isogedunin. Results of these molecular modeling approaches are presented in the next section.

## Materials and Methods

### Protein Preparation and Docking Protocol

In order to predict the protein-ligand interactions through molecular docking, known 3D structures of CERT were selected from the Protein Data Bank (PDB) (Burley et al., 2018)^1^. For the docking protocol, the chosen structures were based on a few criteria: (a) The best possible resolution; (b) Protein domains bound to a ligand when available; (c) Protein domains containing no mutations or modified residues; (d) human protein. Accordingly, X-ray crystal structures of START domain complexed with HPA-13 ligand (PDB ID: 3H3Q), unbound PH domain (PDB ID: 4HHV), and START/PH complex (PDB ID: 5JJD) were used as protein targets in the docking protocol (Supplementary Table S1).

### Proteins and Ligands Preparation for Molecular Docking

To have a good estimation of the protonated state of charged residues, each protein was protonated according to the physiological pH (pH = 7.4) using the PROPKA server (Li et al., 2005). About the ligands, ten limonoid compounds reported to inhibit CERT by Hullin-Matsuda et al. (2012) and five (1R, 3R)-N-(3-Hydroxy-1-hydroxymethyl-3-phenylpropyl)alkanamide (HPA) analogs (Kudo et al., 2010) were used in this analysis (Supplementary Figure S1). The molecules were collected in SMILES (Simplified Molecular Input Line Entry Specification) format and converted into 3D conformation using KNIME software (Fillbrunn et al., 2017). Molecules were treated with the same physiological pH (pH 7.4) as the proteins and Gasteiger’s charges were added using an Open Babel script (O’Boyle et al., 2011) before to be saved into mol2 format.

### Molecular Docking Studies

To study the interaction between limonoids/HPAs and CERT domains, water molecules were removed and the interaction surfaces were identified as follow: For START and PH domain, a grid around the HPA binding site in START domain and around the sulfate (SO4) that binds in place for ligand binding in PH domain was built (using the option “center on ligand” in AutoDock), respectively. For the START-PH complex, a grid that encompasses the interaction surface between the two domains were developed (using the option center on macromolecule). Then, docking was run with the standard AutoDock (v4.2) suit incorporated in MGL tools (v1.5.6) using Lamarckian Genetic Algorithm (Forli et al., 2016). To identify the most favorable binding site of each inhibitor into the two domains and the complex system, flexible ligand docking was performed. The input grid parameter files were modified and the grid size was adjusted with 0.375 Å grid spacing to cover the active site region of receptors (Supplementary Figure S2). Since the target-ligand poses are ranked using an energy-based scoring function with AutoDock, only the best pose conformation of docked ligand was saved, visualized and studied with PyMOL (Rigsby and Parker, 2016). Basically, the lower is the energy, the higher is the binding affinity. Hydrogen bonding interactions and distances between the different domains and ligands were visualized and measured using PyMOL. PyMOL was also considered in the development of several virtual mutations of residues known to play an important role in the interaction of PH and START domains (Prashek et al., 2017).

### Stability Evaluation by Molecular Dynamics Simulations (MD)

The MD approach was performed to study the dynamic behavior of CERT as well as the structural stability of START-PH complex with docked isogedunin and HPA-12 ligands. MD simulations were carried out using GROMACS software package (v5.1.4) (Van der Spoel et al., 2005). First, ligand-free CERT topology was prepared using “pdb2gmx” with the OPLS-AA/L all atom force field. On the other hand, PDB files of docked complexes (CERT-isogedunin and CERT-HPA-12) were separated into PDB files of protein and ligand. Then, topology of CERT was prepared using GROMOS96 43a1 force field. The ligands topology was developed using the “PRODRG” server (van Aalten et al., 2005). The box (unit cell) in which the protein was located has been defined and the system has been filled with water. The protein ligand system was kept in a cubic box, filled of waters (TIP3) and preserving a minimum distance of 10 Å between each atom of the system and walls of the box. The resulting system was solvated by a single point charge (SPC) 216 solvent model, provided in GROMACS parameters. In order to neutralize the system with a net charge of -7, counter ions of 7 NA + were added. An energy minimization was performed on the system to eliminate steric clashes using the “steepest descent” method. Next, the system was equilibrated for 100 ps with 50,000 steps. For the equilibration phase, NVT (Number of particles, Volume, and Temperature) equilibration was performed for 100 ps at a temperature of 300k with a coupling constant of 0.1 ps. Once the temperature was stabilized, NPT (Number of particles, Pressure, and Temperature) was run by setting the temperature to 300k and the pressure to 1 bar. Electrostatic interactions were calculated using the Particle-Mesh Ewald method (PME). Finally, the production phase was performed for 50 ns (MD run) with a time step of 2 fs to make sure the system is stable. The MD simulation was run in triplicate on each system. The parameters of the MD simulations are described in Supplementary Figure S3.

### MM-PBSA Analysis

The molecular mechanics Poisson Boltzmann surface area (MM-PBSA) method is the widely used method for binding free energy calculations from the snapshots of MD trajectory and *g_mmpbsa* was used for this present study (Kumari et al., 2014). It integrates functions from GROMACS and APBS^2^ to determine the polar and non-polar contributions of the binding energy. The dielectric relative constant ε has been set to 2 for ligands and 80 for water (Kukic et al., 2013). In this approach, the binding free energy Δ Gbind between a protein and a ligand include different energy terms and could be calculated as:

Δ⁢Gbind=Gcomplex-(Gprotein+Gligand)

=Δ⁢EMM-T⁢Δ⁢S+Δ⁢Gsol

=Δ⁢Evdw+Δ⁢Eele+Δ⁢GPB+Δ⁢GSA-T⁢Δ⁢S

Δ Gbind is the binding free energy. Δ EMM stands for the gas-phase interaction energy, which is the sum of van der Waals energy Δ Evdw and electrostatic energy Δ Eele. Δ Gsol is the sum of polar solvation energy Δ GPB and the non-polar solvation energy Δ GSA. The polar solvation energy is calculated using Poisson Boltzmann (PB) approximation model, while the non-polar solvation energy is estimated by solvent accessible surface area (SASA). The entropy contribution (−TΔ S) is ignored in this study because of its expensive computational demand. MM/PBSA is applied to, the 50 ns MD simulations (500 ps-spaced) of our different protein-ligand systems to estimate their free binding energies.

## Results and Discussion

### Docking Analysis of START- PH- and START-PH Complex Ligands Interactions

In this study, fifteen natural compounds were docked into each of the three different domains, i.e., START and PH domains independently (Table 1), and on the START/PH interaction domain (Table 2). We selected the top binding pose of each molecule bound to the domains, based on the binding energies estimated by AutoDock (Onawole et al., 2018).

**TABLE 1:** Docking results of the 15 ligands on PH domain and START domain.

	PH	START

Ligands	Energy (kcal/mol)	Energy (kcal/mol)
Carapin	–6.27	–8.48
Cedrelone	–7.18	–8.62
Isogedunin	–6.85	–9.79
Khayantone	–6.71	–10.17
Khivorin	–6.61	–8.53
Limonin	–6.95	–6.26
Methyl angolensate	–6.99	–8.07
Obliquin	–5.58	–6.06
Odoratone	–6.81	–10.60
Prieuranin	–2.61	–3.28
HPA-12	–4.40	–6.57
HPA-13	–4.93	–6.85
HPA-14	–4.73	–7.11
HPA-15	–4.83	–6.71
HPA-16	–3.07	–6.79

**The energy score is a binding affinity estimation between a compound and the surrounding residues.**

**TABLE 2:** Docking results of the 15 ligands on the START-PH interaction site wild type and on 4 CERT mutants.

Ligands	PH-START interface				
	
	WT	R43A	R43A/Y54A/W33A	E494R/N495K	E494R/N495K/P535R/E537K

	Energy (kcal/mol)	Energy (kcal/mol)	Energy (kcal/mol)	Energy (kcal/mol)	Energy (kcal/mol)
Isogedunin	–11.75	–12.03	–11.08	–11.31	–11.38
Cedrelone	–9	–8.91	–8.71	–8.88	–8.86
Limonin	–9.93	–9.95	–9.53	–10.24	–10.23
Khivorin	–5.91	–1.43	–0.95	0.32	–2.73
Khavanthhone	–3.74	–3.34	–2.18	–3.31	–3.13
Obliquin	–6.93	–6.93	–7.05	–6.94	–6.88
Odoratone	–7.55	–6.74	–10.56	–7.17	–6.85
Methyl angolensate	–9.65	–9.74	–9.63	–10.29	–10.21
Prieuranin	0.14	1.19	14.93	10.38	8.99
Carapin	–8.66	–8.27	–8.62	–8.6	–8.5
HPA-12	–6.99	–8.17	–7.64	–7.88	–7.48
HPA-13	–5.62	–7.46	–8.11	–7.83	–7.59
HPA-14	–6.36	–8.91	–7.21	–8.91	–7.56
HPA-15	–5.85	–6.8	–7.74	–7.25	–6.52
HPA-16	–6.45	–6.83	–6.68	–6.8	–8.4

**The energy score is a binding affinity estimation between a compound and the surrounding residues. The mutants R43A, Y54A, and W33A are located in the PH domain, whereas E495R, N495K, P535R, and E537K are located on the START domain.**

**FIGURE 2:**
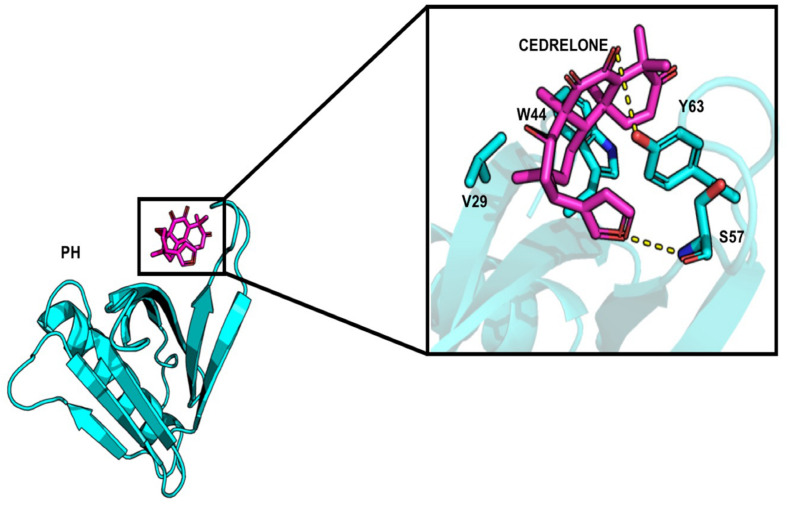
Representation of cedrelone docked into the PH domain. Cedrelone is in magenta and the residues interacting with cedrelone are in sticks.

About the PH domain, it was reported that the SO4 ligand bound with R43, K32, Y54, and K56 (Prashek et al., 2013). In our study, most of the docked ligands do not interact simultaneously with these four residues but only with one of them. Cedrelone was the ligand with the lowest binding energy, with an estimated binding score of −7.18 kcal/mol (Table 1). Cedrelone formed two hydrogen bonds with Y63 and S57 located on loop β3/β4. This loop is known to contribute to the conservative PH domain pocket composition. Additional contacts including hydrophobic interactions, have been observed with residues W44 and V29 (Figure 2). In opposite, all the HPA’s analogs bound with higher energies than the limonoids compounds (i.e., lower binding affinity) (Table 1). They preferably interacted with hydrophobic residues and formed hydrogen bonds with the residue R43 only. These results suggest a more suitable interaction site for limonoids compounds into the PH domain.

**FIGURE 3:**
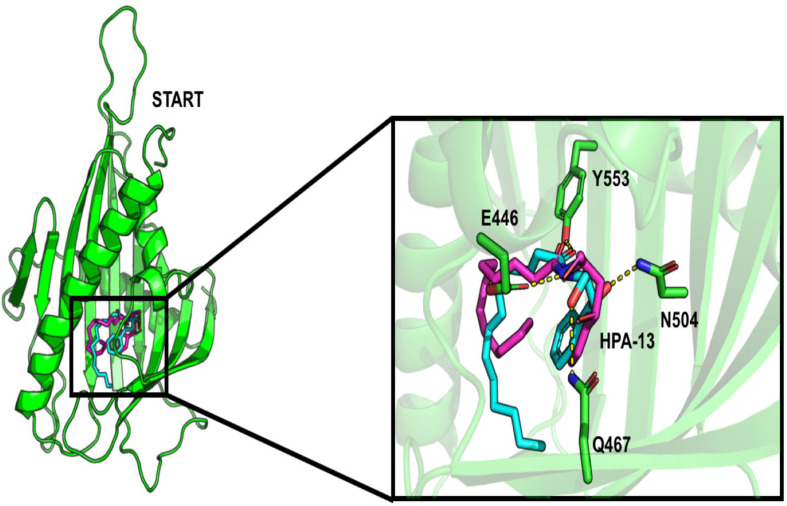
Comparison of the interaction of HPA-13 into the START domain from the X-ray (in cyan) and the docking approach (magenta). Residues that interact with both poses are in sticks.

About the START domain, HPA-13 has been reported to bind with residues N504, E446, Y553, and Q467 (Kudo et al., 2010). From our docking analysis, HPA-13 did not recover completely the same pose as the one observed in the crystal structure (Figure 3). This is probably due to the long alkyl chain which gives some flexibility to the molecule. Still, interactions with residues E446, Q467, and Y553 are present.

**FIGURE 4:**
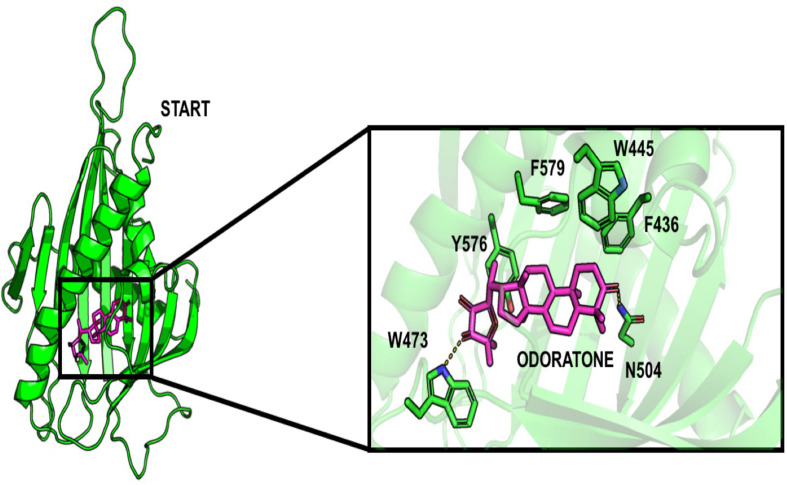
Docking pose of odoratone into the START domain. The odoratone is in magenta and the residues of START domain surrounding the ligand are in green. The odoratone and the residues involved in the ligand binding are represented in sticks.

Surprisingly, some limonoids compounds showed lower binding energies than HPA compounds suggesting that these compounds would have a higher inhibition effect than HPA ligands on the START domain (Table 1). For example, carapin forms hydrogen bonds with Y553, N504, Q467, and E446. Hydrophobic interactions are made with W445, T448, I523, V525, and Y576 and a salt bridge with R442 is also present (Figure 4).

**FIGURE 5:**
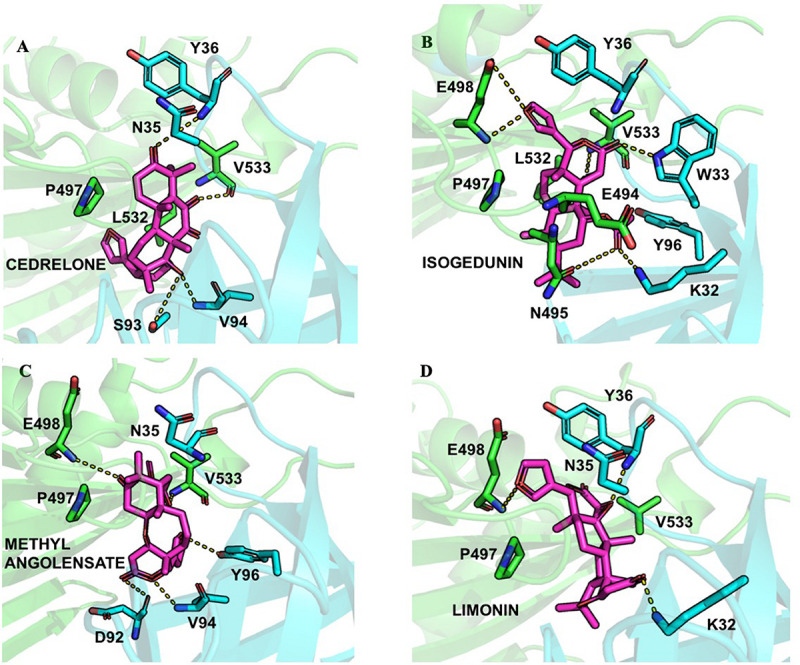
Ligand interactions depicted between **(A)** Cedrelone, **(B)** Isogedunin, **(C)** Methyl angolensolate, and **(D)** Limonin within the PH-START interface.

Finally, following the study from Prashek et al. (2017) ligands were docked into the START-PH interaction domain in order to evaluate if these compounds could have an impact by stabilizing the inhibitory mechanism of interaction between these two domains. Again, limonoid compounds showed lower binding energies score to START-PH compared to the HPA ligands. Isogedunin, cedrelone, and methylangolensate have the lowest binding energies when the two domains are linked together, resulting in more favorable interactions (Table 2). About isogedunin, hydrogen bonds are made with N495, P497, E498 from the START domain, and with W33 and Y35 from the PH domain. Hydrophobic interactions can be observed with Y96, P497, and L532. Furthermore, isogedunin forms a salt bridge with K32, as well as π-stacking with Y36. All these interactions seem to stabilize isogedunin at the interface of CERT. Interactions with some of these residues are found for other limonoids such as limonin, methyl angolensate, and cedrelone and represented in Figure 5.

Interestingly, some of the residues that participate in the binding with these ligands have recently been reported to be important in the interaction of the two domains. Residues of the START domain, such as N495, E498, and V533, appeared to form extensive hydrogen bonds, and hydrophobic or π-stacking interactions with K32, W33, Y36, and Y96 which are located at the PH domain. Therefore, we decided to run docking of our set of ligands on four mutated START-PH complexed systems, including the mutations R43A, W33A/R43A/Y54A in PH domain, E494R/N495K and E494R/N495K/P535R/E537K in START domain. For, the limonoid compounds, the mutations do not have a big impact on the binding energies in the interface of the START-PH domains which seems to be maintained in an inactive form. This is not the case for HPA compounds for which docking results reveal mutations improve the ligands’ binding affinity for many HPA compounds (Table 2). However, the binding energies are still weaker than with some limonoid compounds such as isogedunin, limonin, or methyl angolensate. At the end of this docking study, we could conclude that the limonoid compounds bind more favorably to the interface of the START-PH domains and so could play a major role in the inhibitory activity of CERT by stabilizing this interaction. To estimate further this hypothesis, we decided to run several molecular dynamic (MD) simulations corresponding to (i) the START-PH complex without ligand and with different mutations suggested previously, (ii) the START-PH complex with the HPA-12 ligand, (iii) the START-PH complex with the isogedunin ligand, (iv) the START-PH complex with the mutation E494R/N495K and the isogedunin ligand. For the last one, the E494R/N495K mutant was reported to be important to the PH-START interaction and is also present in the binding of isogedunin. So, it is expected to observe how the ligands are stabilized during the MD simulations.

### Stability Evaluation of PH-START Complex by MD Simulations

#### Backbone RMSD of the Wild Type and Mutated PH-START Complex

Based on the 50 ns of production from the MD simulations for the WT and the 4 CERT mutants, root-mean square deviation (RMSD) analysis enabled the measure of average distances (in Å) of the studied systems from the corresponding starting structure over the simulation period. We have to notice that the G387 residues on the START domain was missing in the X-ray structure. Therefore, the region from the N terminal T364 to the C terminal of V386 was removed from the RMSD and RMSF analysis as it was fluctuated much more compared to the others area.

**FIGURE 6:**
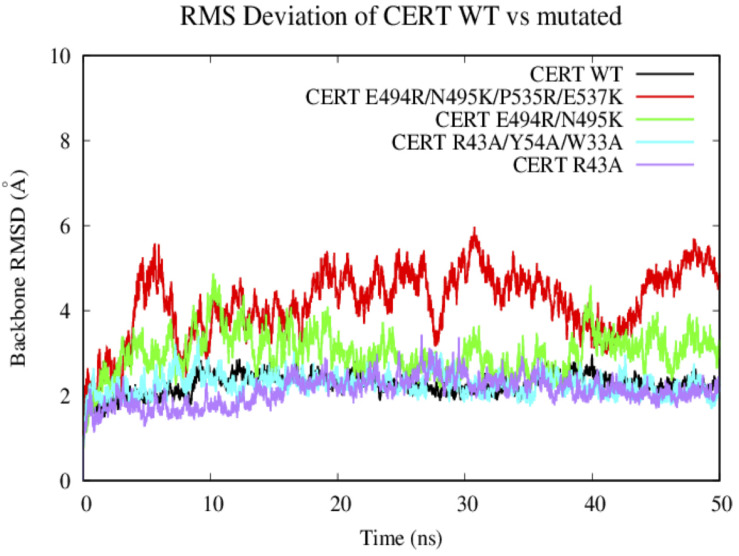
Evaluation of the RMSD (Å) of the wild-type and mutated protein as a function of the simulation time (50 ns).

In general, the systems were relatively stable with a maximum RMSD around 4 Å. The WT and the two other CERT mutants on the PH domain (R43A and W33A/R43A/Y54A) remain stable around 2 Å along the 50 ns. These mutants seem to destabilize the interaction of the START-PH domains less (Figure 6) compared to the CERT mutants, E494R/N495K and E494R/N495K/P535R/E537K, on the START domain. These results suggest that the mutations on the START domain could have an impact on the stability of the START-PH interaction (Prashek et al., 2017).

#### C-Alpha RMSF of the Wild Type and Mutated PH-START Complex

**FIGURE 7:**
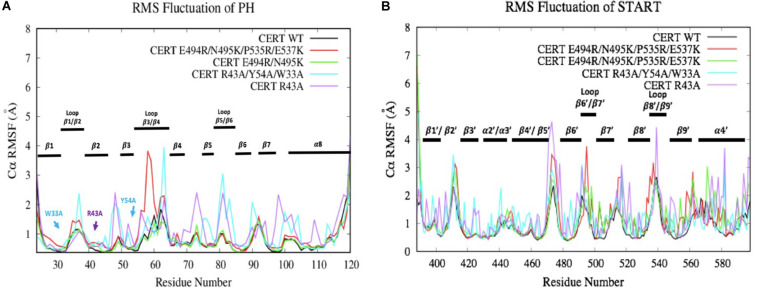
Evaluation of wild-type and mutated PH **(A)** and START **(B)** RMSF (Å) by residue numbers. The mutated residues are indicated by arrows.

The RMSF results are subsequently correlated with the root-mean-square fluctuation of the residues along the MD simulations (RMSF) (Figure 7). Some mutations have more or less affected the flexibility of the PH domain residues. R43A and R43A/Y54A/W33A mutations have significantly increased the flexibility of the loops between the different β-strands. For example, R43A/Y54A/W33A mutation had a greater effect on the β1/β2, β3/β4, and β5/β6 loops which are involved in the interaction surface of both domains. This means that these mutated residues destabilized the environment of the residues on the PH domain surface. However, no effect was observed with the presence of the R494R/N495K mutation. With the E494R/N495K/P535R/E537K mutation, only residues involved in loop β3/β4 (E58-D59) showed an important flexibility with an RMSF value going from 1 to about 4 Å. The START domain showed significant increase in the flexibility of the various loops. A major gain of flexibility is observed on the loop between α1’ and β1’/β2’. Such result was expected since this loop is partly crystallized and therefore tends to be unstable. More importantly, all mutations affected the residues of loops β6’/β7’ and β8’/β9’ involved in the CERT interface and known to be crucial for PH/START stability. Due to the E494R/N495K/P535R/E537K mutation, RMSF values of loop β6’/β7’ went from 2 to 4 Å and this probably explains the instability of the complex. These results also agree with the findings discussed in the previous section.

### Analysis of the Ligands Effect on the PH-START Complex by MD Simulations

Docking approaches help to determine potential ligand binding patterns inside a protein, but such methods do not propose information on the structural stability of protein-ligand complexes. For this reason, in the aim to reinforce the potential mode of binding of limonoid compound at the interface of the START-PH domains, MD simulations were produced (50 ns) and analyzed to evaluate the stability of isogedunin and HPA-12 in the START-PH interaction domains.

#### a. Backbone RMSD and C-Alpha RMSF of CERT Structure

**FIGURE 8:**
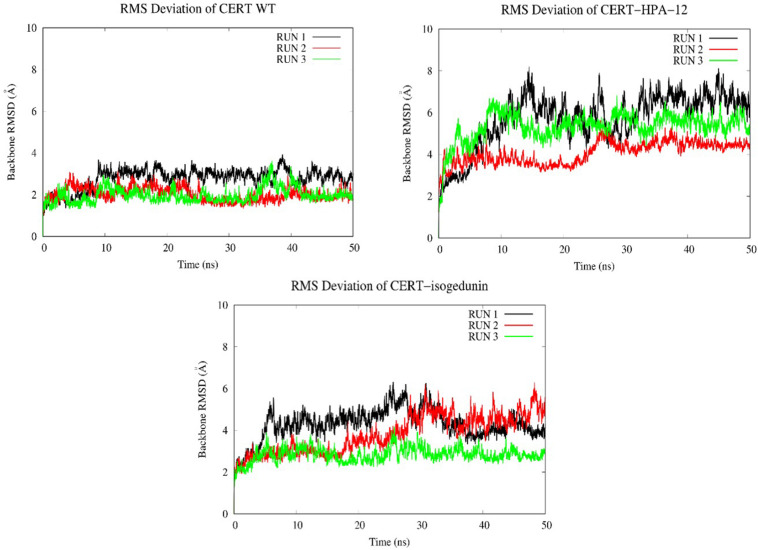
Evaluation of the RMSD (Å) of CERT with and without ligands as a function of time (50 ns) with the protein as a function of the backbone.

Looking at the RMSD measured on the backbone of the two domains, the introduction of the ligands HPA-12 and isogedunin seems to have slightly disturbed the general conformation of the complex system compared to the wild type. We reached in average a RMSD around 2, 4, and 6 Å for the WT and with the introduction of isogedunin and HPA-12 ligands, respectively, (Figure 8). The increase of the RMSD could suggest several dynamic movements around the protein, notably at the interface of the START-PH domains. Isogedunin is relatively more stable compared to HPA for which the RMSD varies sensibly from one to another MD simulation and always higher than for isogedunin. This variability can be explained by the fact that HPA-12 has a very flexible and unstable alkyl chain, as compared to isogedunin that remains more static between the START-PH domains.

**FIGURE 9:**
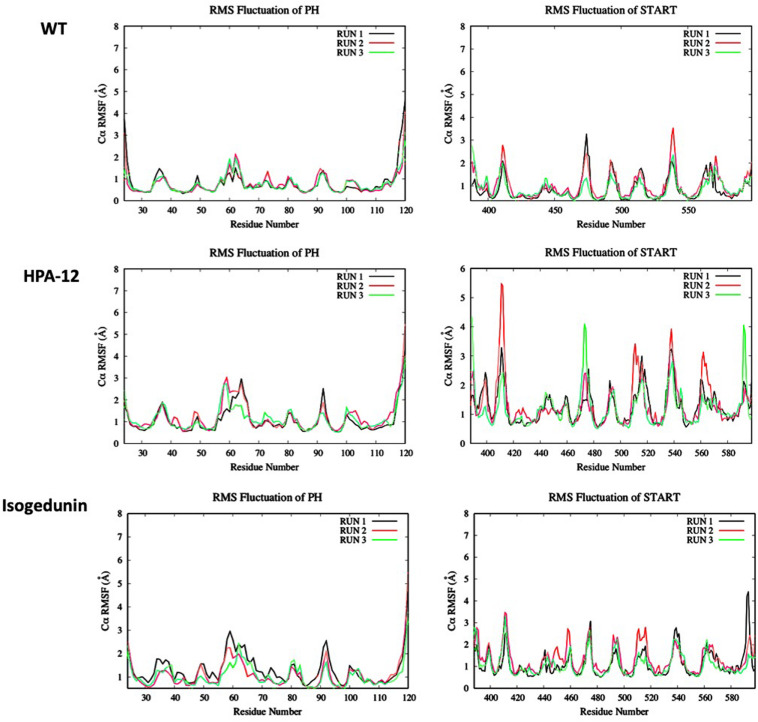
Evaluation of the RMSF (Å) of CERT with and without ligands as a function of time (50 ns) with the protein as a function of the backbone C-alpha.

Looking at the RMSF (Figure 9) of CERT, there are bigger fluctuations in the START domain compared to the PH domain, notably the β’2/β’3 loops and β’5 to β’9 and α’4/β’8. A similar trend is observed with isogedunin except that the fluctuations are a little higher in α3 and β5/β6 loops in the PH domain and β’5/β’6 in the START domain. In opposite, the RMSF are much higher for HPA-12, especially in the START domain suggesting that HPA-12 is more disturbing the START-PH complex than isogedunin.

**FIGURE 10:**
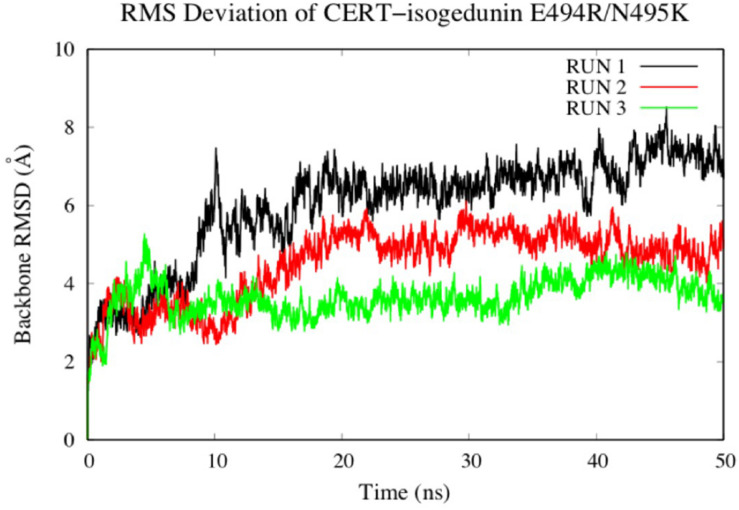
Evaluation of the RMSD (Å) of CERT complexed to isogedunin as a function of time (50 ns) with the protein as a function of the backbone.

To look at the impacts of the E494R/N495K mutation on the CERT-isogedunin complex, three molecular dynamics runs of 50 ns were launched following the same MD protocol (Figure 10). The three runs converged to different states of the complex. In fact, the third run seems be more stable than the two first ones during the simulation time. However, we can clearly notice an increase of the backbone RMSD values compared to the wild type complex. Altogether, the molecular dynamics simulations of the mutated CERT-isogedunin complex reveal more variability. Therefore, we can assume the E494R/N495K mutation might destabilize the CERT-isogedunin complex and disturb their interaction over time.

**FIGURE 11:**
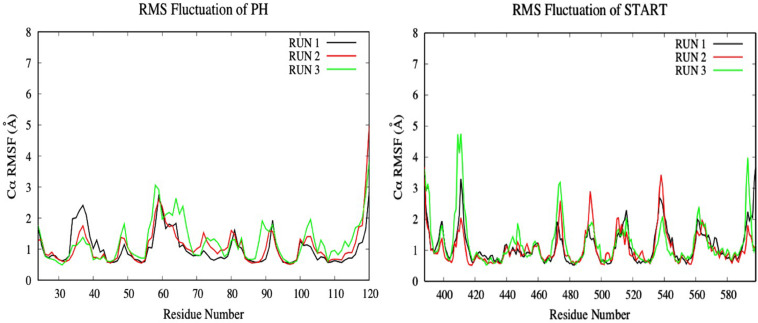
Evaluation of the RMSF (Å) of CERT complexed to isogedunin as a function of time (50 ns) with the protein as a function of the backbone C-alpha.

To investigate the impact of the E494R/N495K mutation on the flexibility of CERT-isogedunin complex, the RMSF of both CERT domains were analyzed (Figure 11). Compared to the wild type complex, only a small increase of the RMSF values were seen. This increase was visible on the main loops involved in the CERT interaction surface. In fact, there is a slight gain of flexibility concerning the β6’/β7’ and β8’/β9’ loops of the START domain and loop β1/β2 loop of the PH domain. These observations show that the E494R/N49RK mutation tends to disturb the START-PH interaction through less stable loops movements on the CERT interface.

#### Hydrogen Bond Analysis

**FIGURE 12:**
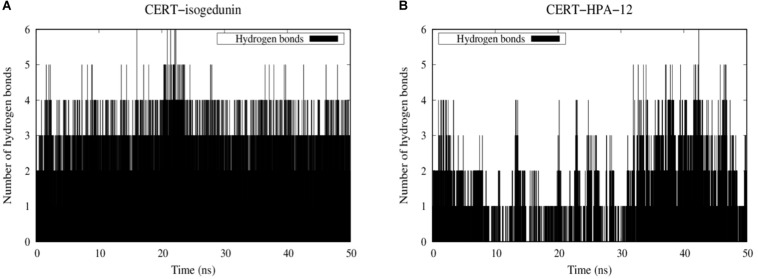
Evolution of stability of CERT-isogedunin **(A)** and CERT-HPA-12 **(B)** complexes using a diagram showing intermolecular hydrogen interactions as a function of time (ns) with Gromacs (Van der Spoel et al., 2005).

Hydrogen bonds are known to play an essential role in the molecular recognition and stability of protein structures. A greater number of interactions between the intermolecular hydrogen bond interaction results in the better stability of the protein-ligand complex. In this study, a hydrogen bond analysis was conducted to examine the stability of docked isogedunin and HPA-12 systems. Concerning the CERT-isogedunin system, the hydrogen bond interactions reach a maximum number of 6 between 20 and 21 ns. Afterward, the hydrogen bonds numbers remain stable throughout the MD simulation and only fluctuate between 3 or 4 (Figure 12A). The hydrogen bond interactions number of CERT-HPA-12 system reaches a maximum of 6 as well but only up to 42 ns, then drops significantly to one and comes back to two at the end of the simulation. Furthermore, major interruptions dropping the number of bonds to 0 were observed from 9 to 30 ns (Figure 12B). Therefore, we suggest that the few H-bonding of CERT with HPA-12 may enable its disassociation.

To further investigate the stabilization of our systems, trajectories of protein-ligand complexes were analyzed. Hydrogen bonds are constantly formed with Q498 and V533 and isogedunin (Supplementary Figure S4). π-stacking interaction is also present between Y36 and isogedunin along the MD simulation suggesting stability of the ligand at the interface of the START-PH domains. Nonetheless, HPA-12 remained very unstable and flexible on the CERT interaction surface (Supplementary Figure S5). At 10 ns, unlike isogedunin, HPA-12 did not form any hydrogen bonds with CERT. It was maintained at the interface through hydrophobic interactions instead. At 40 ns, it reaches its maximum hydrogen bonds number and interacts with K425, P535, Q537, and R85. However, these bonds quickly break and only K425 remains bonded to HPA-12. More about the contact frequency between residues and ligands are described in Supplementary Information (Contact Frequency Analysis and Supplementary Figure S6).

#### MM-PBSA Analysis

The MM-PBSA calculation of isogedunin and HPA-12 was performed using the g_mmpbsa tool (Table 3). The affinities of the CERT-ligand complexes were analyzed based on the MD trajectory of each system. According to g_mmpbsa calculations, HPA-12 binds to CERT with a Δ Gbind of −168.869 kJ/mol. On the other hand, isogedunin binds to CERT with a lower free energy value of −211.753 kJ/mol. CERT-isogedunin in presence of the E494R/N495K mutation showed a Δ Gbind of −192.407 kJ/mol which is a little higher than the CERT-isogedunin. According, to the energy composition, van der Waals energy seems to be the major force of the binding process of both complexes, with a slightly high affinity for the CERT-isogedunin complex. In fact, van der Waals energy is represented by hydrophobic interactions which play a major role to form stable complexes. The rest of the energy terms, such as the electrostatic energy or the solvent-accessible surface area (apolar) were also favorable components for the binding energy contribution. The overall binding free energies displayed that isogedunin has a better free energy for CERT compared to HPA-12 which is in agreement with previous analyses.

**TABLE 3:** Binding free energy of CERT-HPA-12 and CERT-isogedunin using g_mmpbsa method.

Complexes	Binding energy components (kJ/mol)				
	
	Δ Evdw	Δ Eelec	Δ Epolar	Δ Eapolar	Δ Gbind
CERT-HPA-12	−285.4517.68	−49.0317.30	159.8923.18	−21.271.14	−168.8621.31
CERT-isogedunin	−286.5114.28	−31.2846.63	126.2821.87	−20.241.11	−211.7522.64
CERT-isogedunin E494R/N495K	−263.0455.69	−23.4667.47	112.2634.83	−18.173.37	−192.4132.90

## Discussion

In this study, the interaction between CERT and its potential inhibitors (limonoids and HPAs), as well as the effect of mutated key residues, were investigated using several computational approaches such molecular docking and MD simulations. The docking results allowed us to conclude on the greater affinity of limonoids for the START-PH complex as compared to HPAs. This specific type of compound has a better affinity at the START-PH interface. Mutations conducted on both domains confirmed that the mutated residues have no effect or increase the stability of the interaction between the START and PH domain. The MD simulations and the results of structural characteristic features such as RMSD, RMSF, and hydrogen bonding plots revealed higher stability of isogedunin at the START-PH interface due to increased hydrogen bond interactions. This may imply that this interface would be a favorable site of binding for isogedunin and could explain the assumption of a different inhibitory mechanism of CERT mediated by HPA and limonoid families. At the difference of HPA-12, limonoid compounds are unable to inhibit intracellular transport of fluorescent short chain ceramide from endoplasmic reticulum (RE) to the Golgi apparatus (Hullin-Matsuda et al., 2012). The discrepancy between these results could be explained by our data which suggests that limonoids would stabilize CERT in an inactive form (interaction between PH and START domain) unable to take in charge exogenous fluorescent ceramide (usually used to analyze CERT-mediated ceramide transport in cellulo). Limonoids inhibit the CERT-mediated extraction of endogenous ceramide from isolated membranes (Hullin-Matsuda et al., 2012), also probably by stabilizing CERT under its inactive form. In contrast, HPA-12 which preferentially bound START domain free of the PH domain, could easily prevent the binding of exogenous fluorescent ceramide. Prashek et al. have shown that the stability of PH-START domain interaction dictates CERT subcellular localization (Prashek et al., 2017). Indeed, disruption of PH-START domain interaction results in the translocation of CERT from the ER to the Golgi apparatus, mediating the transport of ceramide. We could therefore hypothesize that limonoids, by stabilizing PH-START interaction domain will stick CERT in the ER. On the other hand, HPA-12, which binds START domain, will rather favor a Golgi (or cytoplasmic) localization of CERT. Finally, interaction between PH and START domain contributes to the inhibition of CERT through its SR hyperphosphorylation (Kumagai et al., 2007). Limonoids, by stabilizing the interaction between the PH and the START domains could also favor hyperphosphorylation of the SR motif and can therefore inhibit CERT function.

## Conclusion

In conclusion, our results demonstrate a novel mechanism of inhibition of CERT by limonoid compounds: an interfacial inhibitory mechanism. These inhibitors are thermodynamically more efficient (as evidenced in our study by comparing HPA-12 and isogedunin) and are likely to be more selective than competitive inhibitors (Pommier and Marchand, 2011). We believe that our findings will provide insights in the development of *in vitro* assays that can validate our computational study and guide for the development of limonoid analogs that could selectively target CERT and used in novel cancer therapy strategies.

## Data Availability Statement

The raw data supporting the conclusions of this article will be made available by the authors, without undue reservation, to any qualified researcher.

## Author Contributions

HL, CM, and OT elaborated the study. MG and OT performed the modeling analysis. All authors contributed to the writing of the manuscript.

## Conflict of Interest

The authors declare that the research was conducted in the absence of any commercial or financial relationships that could be construed as a potential conflict of interest.

## Footnotes

1 http://www.rcsb.org/

2 http://rashmikumari.github.io/g_mmpbsa/
