# Supplementary material for: Identification of the Interactions Interference Between the PH and START Domain of CERT by Limonoid and HPA Inhibitors
Source: Front Mol Biosci. 2020 Nov 27;7:603983. doi: 10.3389/fmolb.2020.603983 (PMC7729066; doi:10.3389/fmolb.2020.603983)
Supplement: Supplementary file 1 [file Data_Sheet_1.PDF]

Supplementary information of the manuscript "Identification of the interactions interference between the PH and START domain of CERT by limonoid and HPA inhibitors".

Table 1: Summary table of the protein targets

PDB code	Protein	Reference
5JJD	START/PH complex	Prashek et al., 2017
4HHV	PH domain	Prashek et al., 2013
3H3Q	START domain	Kudo et al. 2010

Figure S1: 2D structure representation of the fifteen ligands analysed in this study.

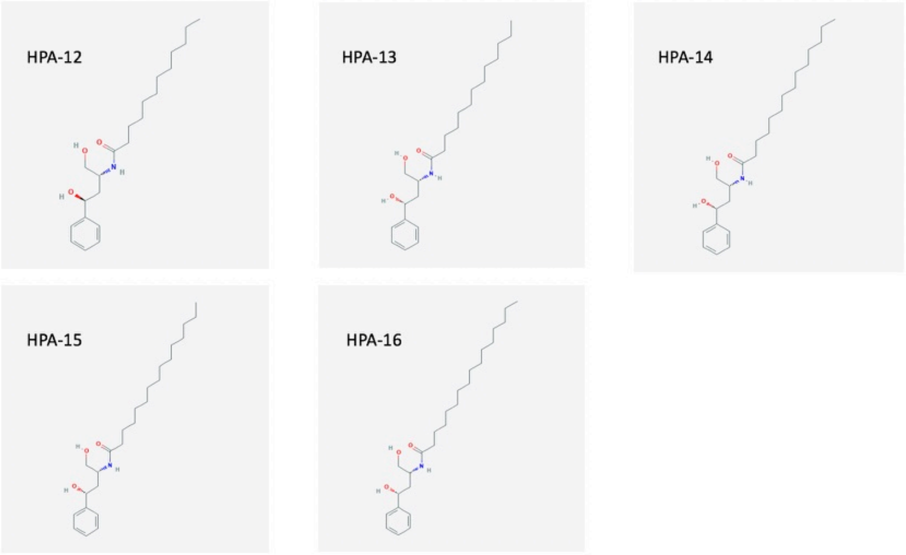

Khayanthone

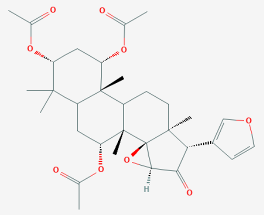

Isogedunin

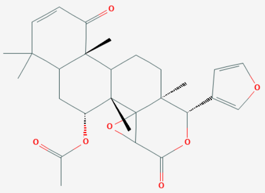

Angolensate methyl

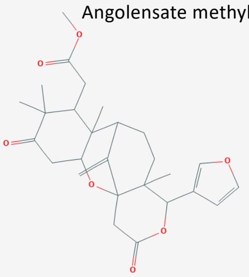

Carapin

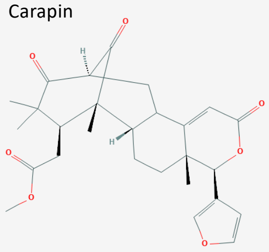

Cedrelone

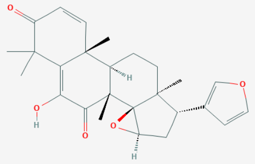

Khivorin

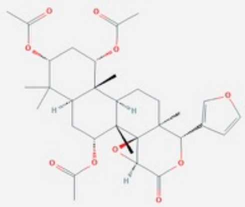

Limonin

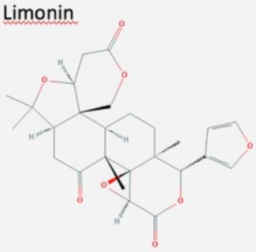

Obliquin

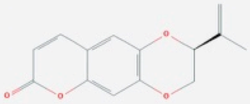

Odoratone

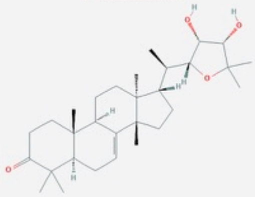

Prieuranin

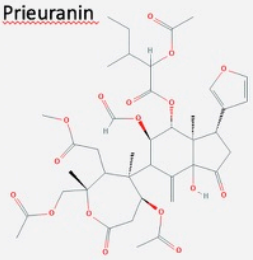

Information about the Autodock 4.2 and parameters used in the study.

AutoDock 4.2 uses a semi-empirical free energy force field to evaluate conformations during docking simulations. The force field was parameterized using a large number of protein-inhibitor complexes for which both structure and inhibition constants, or Ki , are known.

The force field evaluates binding in two steps. The ligand and protein start in an unbound conformation. In the first step, the intramolecular energetics are estimated for the transition from these unbound states to the conformation of the ligand and protein in the bound state. The second step then evaluates the intermolecular energetics of combining the ligand and protein in their bound conformation. The force field includes six pair-wise evaluations (V) and an estimate of the conformational entropy lost upon binding (ΔS_{conf}):

$$\Delta G = (V_{bound\ L-L} - V_{unbound\ L-L}) + (V_{bound\ P-P} - V_{unbound\ P-P}) + (V_{bound\ P-L} - V_{unbound\ P-L} + \Delta S_{conf})$$

where L refers to the “ligand” and P refers to the “protein” in a ligand-protein docking calculation.

Figure S2: Box coordinates and details covering the PH domain, the START domain and the loops involved in the START/PH interface.

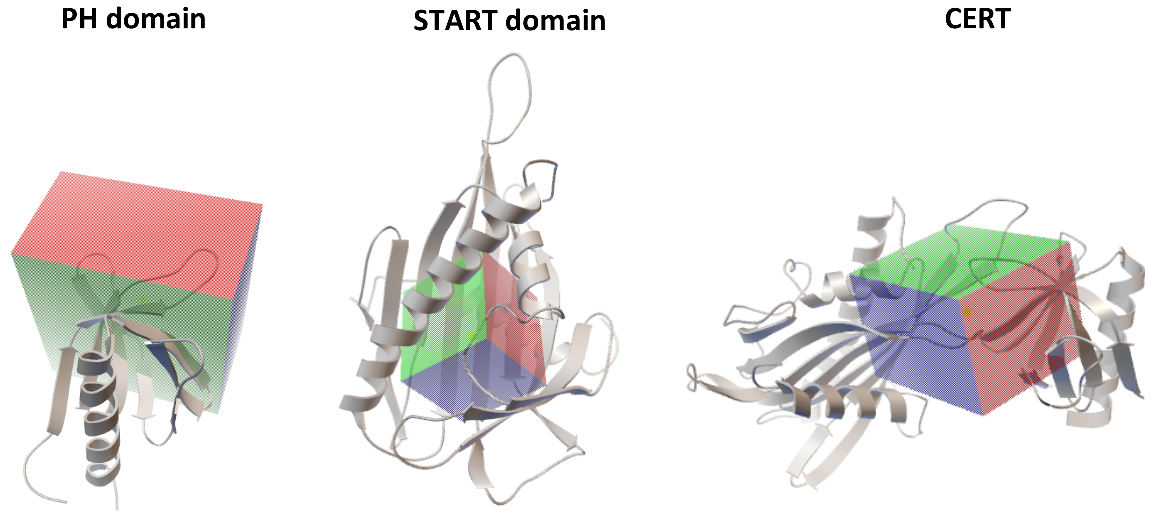

	PH	START	CERT
X center	78	38	66
Y center	46	36	46
Z center	80	42	56
X size	21.689	9.75	-51.245
Y size	5.031	1.186	-5.394
Z size	51.588	33.824	-37.443
Spacing	0.375	0.375	0.375

Figure S3: Molecular Dynamics protocol

The OPLS-AA/L all-atom force field was applied to study the CERT WT and mutated systems. On the other hand, the GROMOS96 43A1 force field, which is more suited for protein-ligand complexes, was applied on the CERT-isogedunin (mutated vs non-mutated) and on the CERT-HPA-12 complexes.

	**CERT with and without mutations**	**CERT with and without ligands**
**Preparation of the structure**	Propka: Protonation of the protein (pH= 7.4)	Propka: Protonation of the protein (pH= 7.4)<br>**Open Babel**: Protonation of ligands and determination of charges (Gasteiger)
**Preparation of the system (GROMACS)**	- Force field = OPLS-AA/L all-atom<br>- Solvation = TIP3P water molecules<br>- Cubic box, distance: 10 Å<br>- Neutralisation of the system: 7 NA+	- Force field: GROMOS96 43A1<br>- Ligand topology: PRODRG 2.5<br>- Solvation: TIP3P water molecules<br>- Cubic box, distance: 10 Å<br>- Neutralisation of the system: 7 NA+
**Minimization**	- Method: Steepest descent<br>- Number of step: 50 000<br>- Fmax = 1000kJ/mol/nm	- Method: Steepest descent<br>- Number of step: 50 000<br>- Fmax = 1000kJ/mol/nm
**Equilibration**	- Time of simulation: 100 ps<br>- System isotherme-isobar (300k, 1 bar)	- Time of simulation: 100 ps<br>- System isotherme-isobar (300k, 1 bar)
**Production**	- Time of simulation: 50 ns<br>- Frames captured every 10 ps<br>- Electrostatic: PME (Particle Mesh Ewald)	- Time of simulation: 50 ns<br>- Frames captured every 10 ps<br>- Electrostatic: PME (Particle Mesh Ewald)

Figure S4: Isogedunin binding patterns with residues at the CERT interaction surface at different time intervals. (**A**) 10 ns, (**B**) 20 ns, (**C**) 30 ns, (**D**) 40 ns and (E) 50 ns.

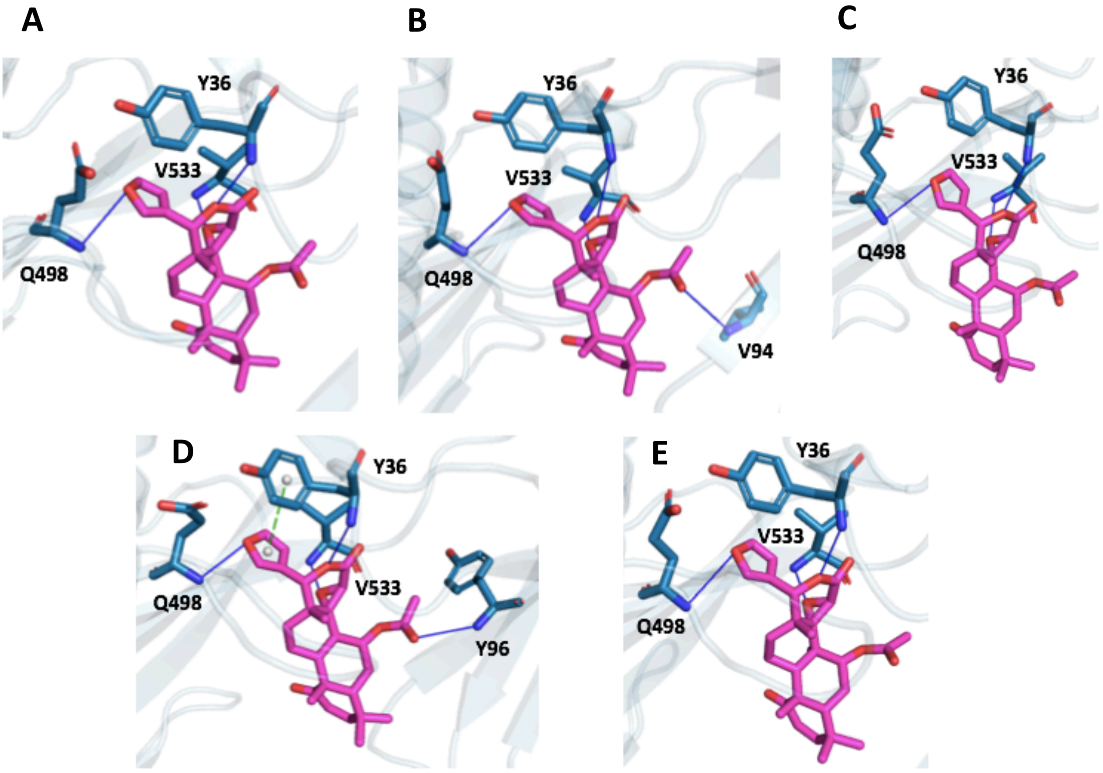

Figure S5: HPA-12 binding patterns with residues at the CERT interaction surface at different time intervals. (**A**) 20 ns, (**B**) 30 ns, (**C**) 40 ns and (**D**) 50 ns.

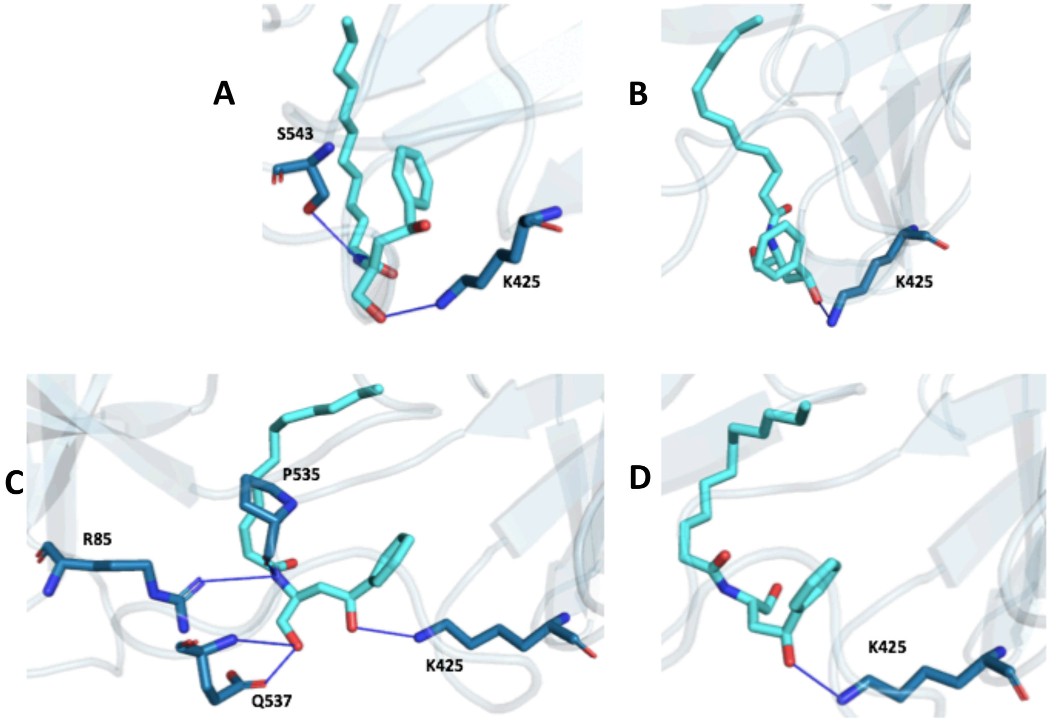

- **Contact frequency analysis during MD simulations.**

A residue-based contact frequency analysis in each MD run of our different systems (CERT WT, CERT-HPA-12, CERT-isogedunin and CERT-isogedunin with E494R/N495K mutation) was performed to identify contact key residues and their sustainability within CERT interaction. Contacts are considered at a 4 Å distance. All of the known contact residues present in CERT interaction surface (Prashek et al., 2017) were recovered in our analysis (**Figure S6A**). Most of these residues (TRP33, TYR36, TRP40, ARG43, TYR54, ARG66, TYR96, E494, N495, VAL533 and PRO535) are shown to have stable and constant contacts through the 50 ns three MD replicas. Therefore, these residues contribute to the stability and to the interaction strength of the START/PH complex. This approach was also applied on the CERT-HPA-12 complex (**Figure S6C**). Only a few residues (E494, N495, ASP496, PRO497, LEU532, VAL533 and SER534), mostly on the START domain, maintained a stable contact frequency in the different MD simulations. The lack of contact frequency explains the instability of the complex and validate the results obtained in the hydrogen bond analysis. Furthermore, we compared the residues contact frequency of CERT-isogedunin complex

with and without the E494R/N495K mutation in order to have an idea of the impact of the mutation on the complex interaction (**Figure S6B and S6D**). Interestingly, the results is in agreement with our previous analyses. Most of the residues on both domains almost reach a perfect contact frequency when the CERT-isogedunin lacks the E494R/N495K mutation. Therefore, the continuous and persistent residues contacts attest the stability of the complex. However, these same residues are slightly inconsistent in the mutated complex and a clear decrease in the contact frequency is observed. Thus, the E494R/N495K mutation do disturb the CERT-isogedunin interaction in addition to the loop's flexibility on the CERT interface. Moreover, isogedunin revealed a more stable and sustained residues contact frequency through the MD simulations compared to HPA-12.

A

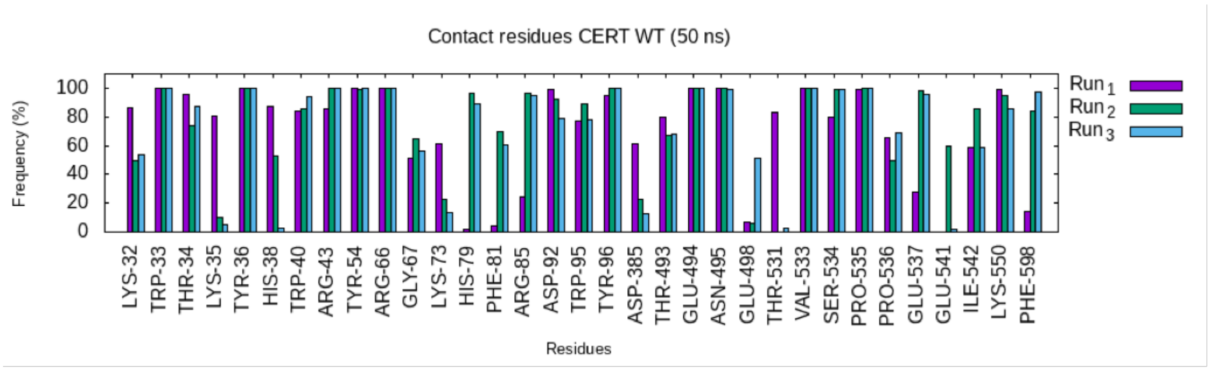

B

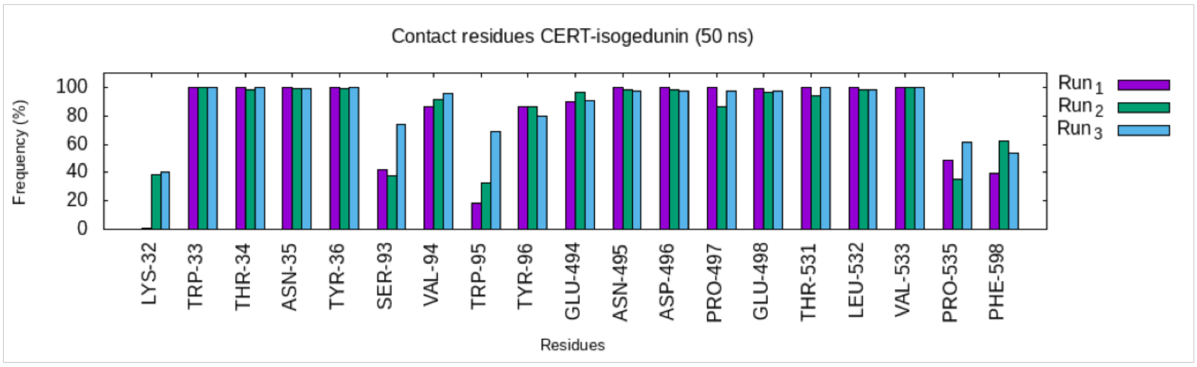

C

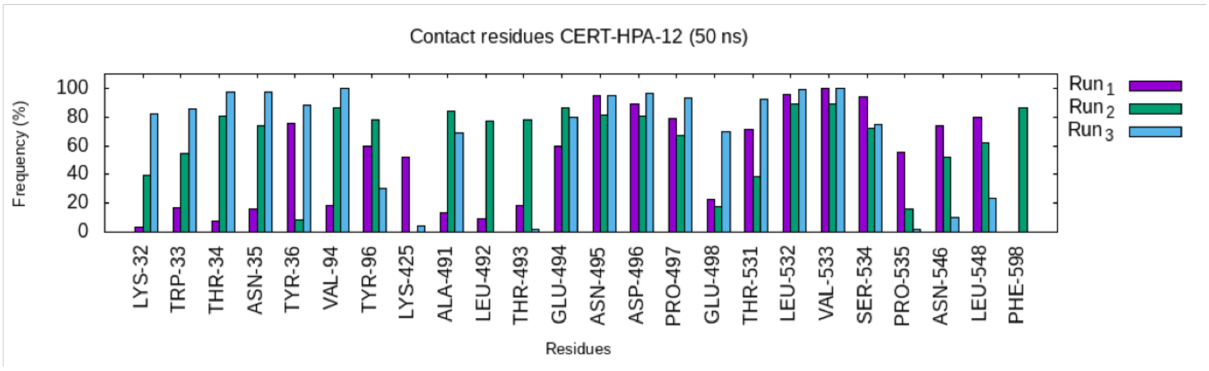

D

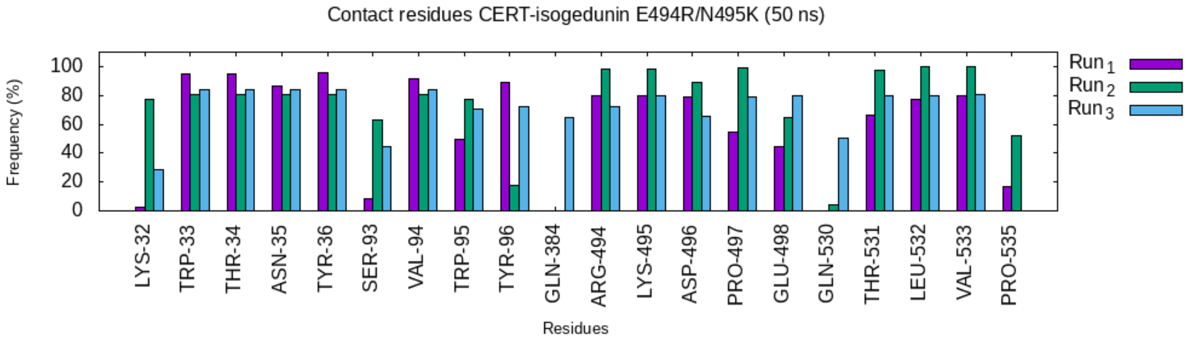

Figure S6: Residue based contact frequency (%) during MD simulations: (A) CERT WT, (B) CERT-isogedunin, (C) CERT-HPA-12, (D) CERT -isogedunin in presence of the E494R/N495K mutations.
